# ARTEMIN Promotes *De Novo* Angiogenesis in ER Negative Mammary Carcinoma through Activation of TWIST1-VEGF-A Signalling

**DOI:** 10.1371/journal.pone.0050098

**Published:** 2012-11-21

**Authors:** Arindam Banerjee, Zheng-Sheng Wu, Peng-Xu Qian, Jian Kang, Dong-Xu Liu, Tao Zhu, Peter E. Lobie

**Affiliations:** 1 Liggins Institute, University of Auckland, Auckland, New Zealand; 2 Hefei National Laboratory for Physical Sciences at Microscale and School of Life Sciences, University of Science and Technology of China, Hefei, Anhui, People’s Republic of China; 3 Department of Pathology, Anhui Medical University, Hefei, Anhui, People’s Republic of China; 4 Department of Pathology, Shanghai Medical College, Fudan University, Shanghai, People’s Republic of China; 5 Cancer Science Institute of Singapore and Department of Pharmacology, National University of Singapore, Singapore, Singapore; Institute of Molecular and Cell Biology, Singapore

## Abstract

The neurotrophic factor ARTEMIN (ARTN) has been reported to possess a role in mammary carcinoma progression and metastasis. Herein, we report that ARTN modulates endothelial cell behaviour and promotes angiogenesis in ER-mammary carcinoma (ER-MC). Human microvascular endothelial cells (HMEC-1) do not express ARTN but respond to exogenously added, and paracrine ARTN secreted by ER-MC cells. ARTN promoted endothelial cell proliferation, migration, invasion and 3D matrigel tube formation. Angiogenic behaviour promoted by ARTN secreted by ER-MC cells was mediated by AKT with resultant increased TWIST1 and subsequently VEGF-A expression. In a patient cohort of ER-MC, ARTN positively correlated with VEGF-A expression as measured by Spearman’s rank correlation analysis. In xenograft experiments, ER-MC cells with forced expression of ARTN produced tumors with increased VEGF-A expression and increased microvessel density (CD31 and CD34) compared to tumors formed by control cells. Functional inhibition of ARTN by siRNA decreased the angiogenic effects of ER-MC cells. Bevacizumab (a humanized monoclonal anti-VEGF-A antibody) partially inhibited the ARTN mediated angiogenic effects of ER-MC cells and combined inhibition of ARTN and VEGF-A by the same resulted in further significant decrease in the angiogenic effects of ER-MC cells. Thus, ARTN stimulates *de novo* tumor angiogenesis mediated in part by VEGF-A. ARTN therefore co-ordinately regulates multiple aspects of tumor growth and metastasis.

## Introduction

Tumor growth and metastasis is dependent on *de novo* angiogenesis. Clinicopathological correlations between angiogenesis and patient survival in mammary carcinoma have been reported [1]. Microvessel density (MVD) was reported to be highest with histopathologically aggressive ductal carcinoma-in situ [1]. High MVD in premalignant lesions has also been associated with high risk of future mammary carcinoma and high MVD has been correlated with metastasis and poor survival in node-negative mammary carcinoma [1]. However, the role of angiogenesis in mammary carcinoma remains controversial as a number of studies have indicated lack of therapeutic efficacy of various anti-angiogenic agents, as tumor re-growth during ongoing treatment may be observed [2], [3]. The reasons for these discrepancies may depend upon several factors such as inhibition of vascular endothelial growth factor (VEGF) promoting endothelial vessel normalisation which may decrease delivery of therapeutic agents, thereby indirectly promoting tumor growth. Alternatively, hypoxia due to vascular paucity upon inhibition of angiogenesis may promote tumor invasion, as evidenced whereupon anti-angiogenic treatment of glioblastoma (GBM) resulted in increased intravasation and metastatic dissemination [3]. Such tumor escape mechanisms may partially explain the lack of therapeutic efficacy of inhibition of tumor angiogenesis in mammary carcinoma. In any case, regardless of the controversies surrounding therapeutic inhibition of angiogenesis in mammary carcinoma, angiogenesis remains an important component of tumor growth and metastasis [1].

ARTEMIN (ARTN) is one member of the glial cell line-derived neurotrophic factor (GDNF) family of ligands [4]. ARTN has previously been demonstrated to be involved in progression of various carcinomas [5], [6], [7] including mammary carcinoma [4]. Increased ARTN expression in mammary carcinoma promotes metastasis [8], radio-resistance (manuscript submitted), chemo-resistance [9], endocrine resistance [10] and also enhances CSC like activity in estrogen receptor negative mammary carcinoma (ER-MC) (manuscript submitted). Interestingly, another neurotrophic factor, nerve growth factor (NGF), also stimulates tumor angiogenesis *in vivo* in mammary carcinoma via the PI3K-AKT pathway [11]. Similarly, ARTN could potentially modulate not only tumor growth and metastasis, but also promote angiogenesis as a contribution to tumor progression leading to poor survival outcomes in ER-MC [8].

The AKT signalling pathway is pivotal to key cellular functions in mammary carcinoma including metastasis and angiogenesis [12]. The expression of various angiogenic factors including VEGF-A and angiopoietins (ANG), and their receptors, are regulated by AKT activity in mammary carcinoma. AKT expression is also correlated with VEGF-A expression and MVD in mammary carcinoma [12]. Furthermore, AKT activation controls the tumor microenvironment by promoting endothelial cell proliferation, survival and migration regulating tumor angiogenesis through VEGF dependent pathways [13]. Thus, AKT plays an important role in the tumor angiogenic process.

The basic helix-loop-helix transcription factor TWIST1, also promotes tumor angiogenesis and metastasis in mammary carcinoma [14]. Previously we have demonstrated that ARTN promoted oncogenicity and invasion is mediated by TWIST1 in ER-MC cells [8]. Promotion of *de novo* angiogenesis in human mammary carcinoma by TWIST1 has been reported [15]. Various pro-angiogenic factors including VEGF-A and ANG and their receptors were demonstrated to be positively regulated by TWIST1 in murine melanoma cell lines [15]. A recent study also suggested that TWIST1 positively regulates VEGF-A mRNA levels in metastatic mammary carcinoma [16]. Furthermore, TWIST1 mediated angiogenesis in mammary carcinoma in clinical samples correlates with higher expression of VEGF-A [14].

We report herein that ARTN secreted from mammary carcinoma cells promotes tumor angiogenesis which is mediated in part by enhanced VEGF-A expression. Thus, ARTN co-ordinately regulates angiogenesis and tumor progression of ER-MC.

## Results

### Paracrine ARTN Modulates HMEC-1 Proliferation, Migration, Invasion and Tube Formation

**Figure 1:**
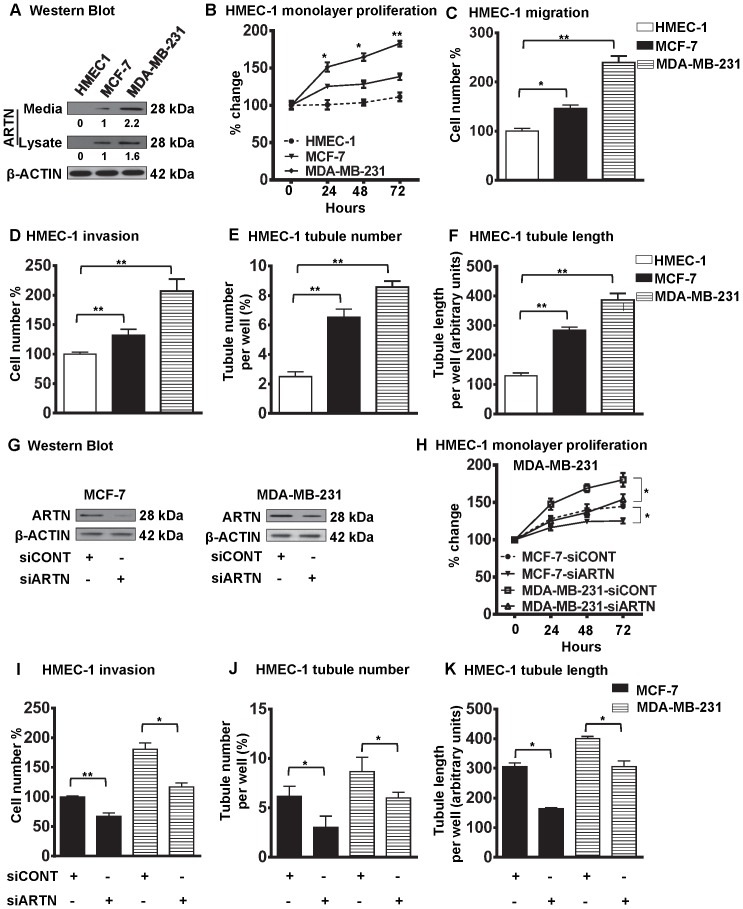
ARTN secreted from mammary carcinoma cells possesses a functional role in modulating endothelial cell behaviour. (A) Western blot analysis for ARTN protein expression in HMEC-1, MCF-7 and MDA-MB-231 cells respectively. Soluble whole cell lysates or concentrated conditioned media were run on an SDS-PAGE and immunoblotted using goat anti-ARTN antibody. β-ACTIN was used as a loading control. (B) HMEC-1 monolayer proliferation. HMEC-1 total cell number after indirect co-culture with either MCF-7 or MDA-MB-231 cells in 2% serum containing media. Cell growth was measured at the indicated time points. Initial numbers of seeded cell are presented as 100%. As an internal control, HMEC-1 total cell number assay without co-culture was also performed. (C) HMEC-1 cell migration after 24 h indirect co-culture with either MCF-7 or MDA-MB-231 cells in serum free conditions. Numbers of migrated HMEC-1 cells without co-culture are presented as 100%. (D) HMEC-1 cell invasion assay after 24 h indirect co-culture with either MCF-7 or MDA-MB-231 cells in serum free conditions. Migratory or invasive cell numbers was calculated as (number of invaded cells through the inserts/total number of cells seeded) × 100. Numbers of invaded HMEC-1 cells without co-culture are presented as 100%. (E) and (F) HMEC-1 cells *in* *vitro* tube formation in matrigel after 12 h indirect co-culture with either MCF-7 or MDA-MB-231 cells in serum free conditions. HMEC-1 tubule number (E) and tubule length (F) was assessed after 12 h. Tubule number was calculated as (number of cells with tubule/total number of cells counted) × 100, whereas tubule length was calculated as an arbitrary units using ImageJ software®. As an internal control, HMEC-1 tubule number and tubule length was also assessed without co-culture. (G) Western blot analyses for ARTN in MCF-7 and MDA-MB-231 cells± siRNA to ARTN. Scrambled RNA was used as siCONT. β-ACTIN was used as loading control for cell lysates. The sizes of detected protein bands in kiloDalton (kDa) are shown on the right. (H) HMEC-1 monolayer proliferation. HMEC-1 cell numbers after indirect co-culture with either MCF-7 or MDA-MB-231 cells in 2% serum containing media ± siRNA of ARTN. Scrambled RNA was use as control. Cells growth was measured at the indicated time points. Initial numbers of seeded cell are presented as 100%. (I) HMEC-1 cell invasion assay after 24 h indirect co-culture with either MCF-7 or MDA-MB-231 cells in serum free conditions ± siRNA of ARTN. HMEC-1 tubule number (J) and tubule length (K) was assessed after indirect co-culture with either MCF-7 or MDA-MB-231 cells ± siRNA of ARTN. *, *p*<0.05; **, *p*<0.01.

To determine the potential role of ARTN in angiogenesis, we investigated the effect of ARTEMIN (ARTN) on proliferation and migration of human microvascular endothelial cells (HMEC-1). We initially choose two different wild type human mammary carcinoma cell lines, MCF-7 and MDA-MB-231 with different endogenous expression levels of ARTN [8] (Figure 1A). Western blot results confirmed that HMEC-1 cells possessed no detectable expression of ARTN protein (Figure 1A). MCF-7 cells exhibited moderate cellular expression; and higher expression and secretion of ARTN protein was observed in MDA-MB-231 cells. During tumor angiogenesis, cellular proliferation and invasion of endothelial cells occurs to generate tumor vasculature [13]. We determined the effects of ARTN secreted by these two mammary carcinoma cell lines on HMEC-1 monolayer proliferation, migration, invasion and tube formation as described previously [17]. The differential expression of the endogenous level of ARTN protein between MCF-7 and MDA-MB-231 cells significantly correlated with HMEC-1 monolayer proliferation. HMEC-1 cell migration, cell invasion, tubule number and tubule length were also increased significantly by co-culture with MDA-MB-231 cells as compared with MCF-7 cells (Figure 1B–F).

To determine the involvement of ARTN secreted from mammary carcinoma cells on endothelial cell angiogenic behaviour we transiently depleted endogenous ARTN expression in MCF-7 and MDA-MB-231 cells by use of siRNA to ARTN [8] (Figure 1G). Scrambled siRNA was used as control. Depletion of endogenous ARTN in MCF-7 or MDA-MB-231 cells resulted in decreased HMEC-1 monolayer proliferation, cell invasion, tubule number and tubule length in MCF-7 and MDA-MB-231 cells as compared to co-culture with the respective MCF-7 and MDA-MB-231 control cell lines (Figure 1H–K). Thus, ARTN secreted by mammary carcinoma cells may possess an important role in modulating endothelial cell behaviour.

### HMEC-1 Cells Express GFRα Isoforms, RET and Respond to Exogenous ARTN Stimulation

**Figure 2:**
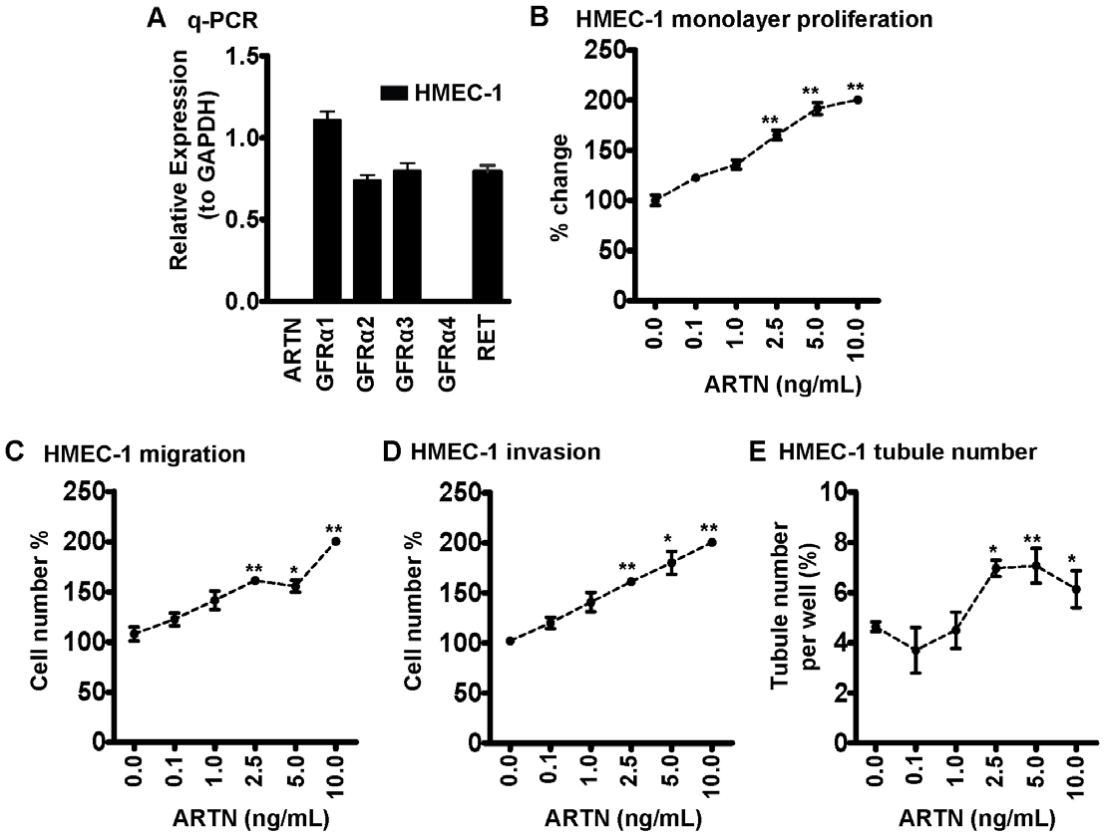
Exogenous ARTN modulates HMEC-1 cell behaviour. (A) The expression of *ARTN*, *GRFα1-4* and *RET* in HMEC-1 cells determined by quantitative real time PCR. Cellular behaviour of HMEC-1 cells, including, (B) Monolayer proliferation. (C) Cell migration. (D) Cell invasion. (E) *in vitro* tubule formation on matrigel were assessed in the presence of different concentration of recombinant ARTN. Recombinant ARTN was added at the indicated concentrations every two days. *, *p*<0.05; **, *p*<0.01.

Previously published work reported that ARTN utilizes GDNF family receptor α3 (GFRα3) or GFRα1 as a ligand binding receptor and RET receptor tyrosine kinase as one common signalling component [18]. Quantitative PCR analysis demonstrated that HMEC-1 cells express endogenous levels of *GFRα1*, *GFRα2*, *GFRα3* and *RET* mRNA, whereas expression of *ARTN* and *GFRα4* was not detectable (Figure 2A). HMEC-1 cells can therefore potentially respond to ARTN.

We next determined whether ARTN exerted direct angiogenic effects by examination of the effect of different concentrations of recombinant human ARTN protein (0.1 ng/mL–10 ng/mL) on HMEC-1 cell function. Exogenously added ARTN significantly stimulated HMEC-1 monolayer proliferation, cell migration, cell invasion and tubule formation in HMEC-1 cells (Figure 2B–E). Thus, ARTN directly modulates endothelial cell behaviour.

### ARTN Secreted from ER-MC Cells Promotes Endothelial Cell Proliferation, Migration, Invasion and Tube Formation *in vitro*

**Figure 3:**
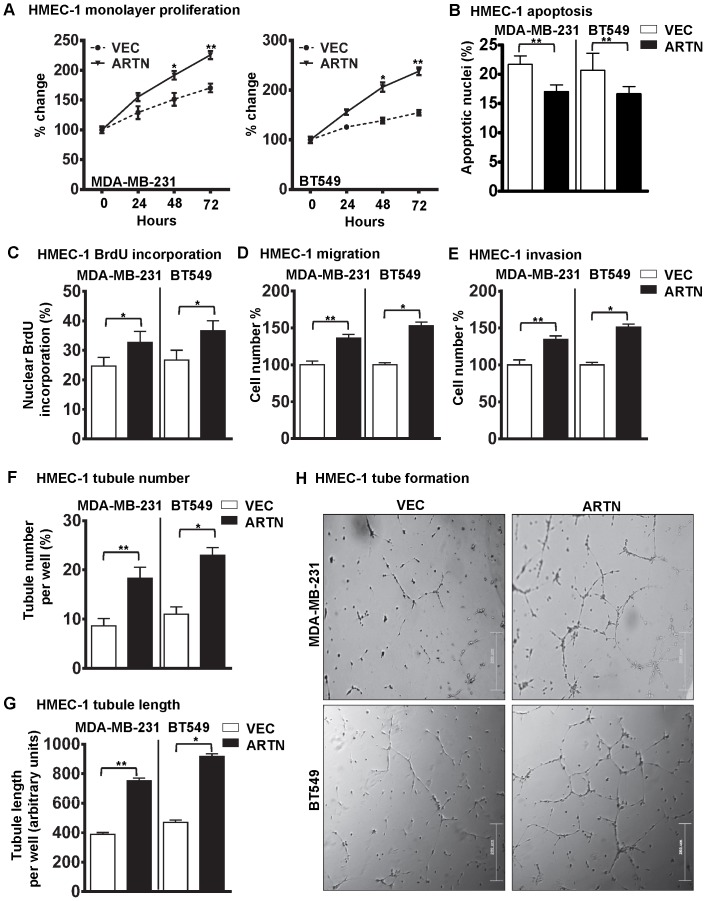
Forced expression of ARTN in ER-MC cells increases the angiogenic potential of HMEC-1 cells. (A) HMEC-1 monolayer proliferation. HMEC-1 total cell number after indirect co-culture with MDA-MB-231 or BT549 cells with forced expression of ARTN in 2% serum containing media. Empty VEC was used as control. Cell growth was measured at the indicated time points. (B) HMEC-1 apoptotic cell death after 24 h indirect co-culture with MDA-MB-231 or BT549 cells with forced expression of ARTN in serum free conditions. (C) HMEC-1 cell cycle progression after 24 h indirect co-culture with MDA-MB-231 or BT549 cells with forced expression of ARTN in serum free conditions. (D) HMEC-1 cell migration after 24 h indirect co-culture with MDA-MB-231 or BT549 cells with forced expression of ARTN in serum free conditions. (E) HMEC-1 cell invasion assay after 24 h indirect co-culture with MDA-MB-231 or BT549 cells with forced expression of ARTN in serum free conditions. (F) and (G) HMEC-1 cells *in* *vitro* tube formation on matrigel after 12 h indirect co-culture with MDA-MB-231 or BT549 cells with forced expression of ARTN in serum free conditions. HMEC-1 tubule number (F) and tubule length (G) was assessed after 12 h. Tubule number was calculated as (number of cells with tubule/total number of cells counted) × 100, whereas tubule length was calculated as an arbitrary units using ImageJ software®. (H) Representative light microscopy images of tube formation. Bar 200 µm.*, *p*<0.05; **, *p*<0.01.

A previous report has suggested that ARTN promotes metastasis of ER-MC cells [8]. Angiogenesis is a crucial component of metastasis, and pro-angiogenic factors, such as VEGF-A [1] produced by the tumor cells have been demonstrated to promote tumor invasiveness, growth and metastasis. Conversely, inhibition of these factors result in decreased tumor growth and metastasis [1]. To specifically examine the role of ARTN secreted from ER-MC cells on HMEC-1 cell function, we used two ER-MC cell lines, MDA-MB-231 or BT549 cells with forced expression of ARTN as previously described [8]. MDA-MB-231 or BT549 cells stably transfected with empty VEC were used as control. Expression levels of both cellular and secretory ARTN proteins are presented in figure S1A–B. The respective cells were co-cultured with HMEC-1 to determine the effects of ARTN secreted by MDA-MB-231 or BT549 cells on HMEC-1 monolayer proliferation (total cell count), invasion, migration and tube formation. Forced expression of ARTN in ER-MC cells increased HMEC-1 monolayer proliferation by 37% and 58% in MDA-MB-231 or BT549 cells respectively as compared to the matched VEC control cells (Figure 3A). Next, we determined the effect of ARTN secreted from ER-MC cells on HMEC-1 cell cycle progression and apoptotic cell death. Indirect co-culture of HMEC-1 cells with MDA-MB-231 or BT549 cells with forced expression of ARTN significantly decreased HMEC-1 apoptotic cell death (22% and 20%) when compared with HMEC-1 cells co-cultured with respective VEC cells (Figure 3B). Analysis of BrdU incorporation demonstrated that co-culture of MDA-MB-231 or BT549 cells with forced expression of ARTN, with HMEC-1 cells, also increased cell cycle progression (26% and 39%) when compared with the respective VEC cells co-cultured with HMEC-1 cells (Figure 3C).

To determine the effects of ARTN secreted from ER-MC cells on the migratory and invasive behaviour of endothelial cells, we performed cell migration and cell invasion assays on HMEC-1 cells co-cultured with either MDA-MB-231 or BT549 VEC or ARTN cells respectively. Indirect co-culture of MDA-MB-231 or BT549 cells with forced expression of ARTN significantly increased HMEC-1 cell migration (39% and 55%) and cell invasion (32% and 53%) over a 24 hr period when compared with respective VEC cells (Figure 3D and E).

Endothelial cells organize when cultured in matrigel and form three dimensional capillary like tubule structures with multiple cell to cell contacts, thus mimicking angiogenesis *in vitro* [17]. To determine the effects of ARTN on endothelial cell tube formation *in vitro*, we performed a tube formation assay with HMEC-1 cells co-cultured with either MDA-MB-231 or BT549 VEC or ARTN cells respectively. Forced expression of ARTN in MDA-MB-231 or BT549 cells significantly increased the tubule number (121% and 136%) and tubule length (91% and 97%) generated by HMEC-1 cells compared with HMEC-1 cells co-cultured with respective control MDA-MB-231 or BT549 cells (Figure 3F–H).

### Depletion of ARTN in ER-MC Cells Inhibited Endothelial Cell Proliferation, Migration, Invasion and Tube Formation *in vitro*

**Figure 4:**
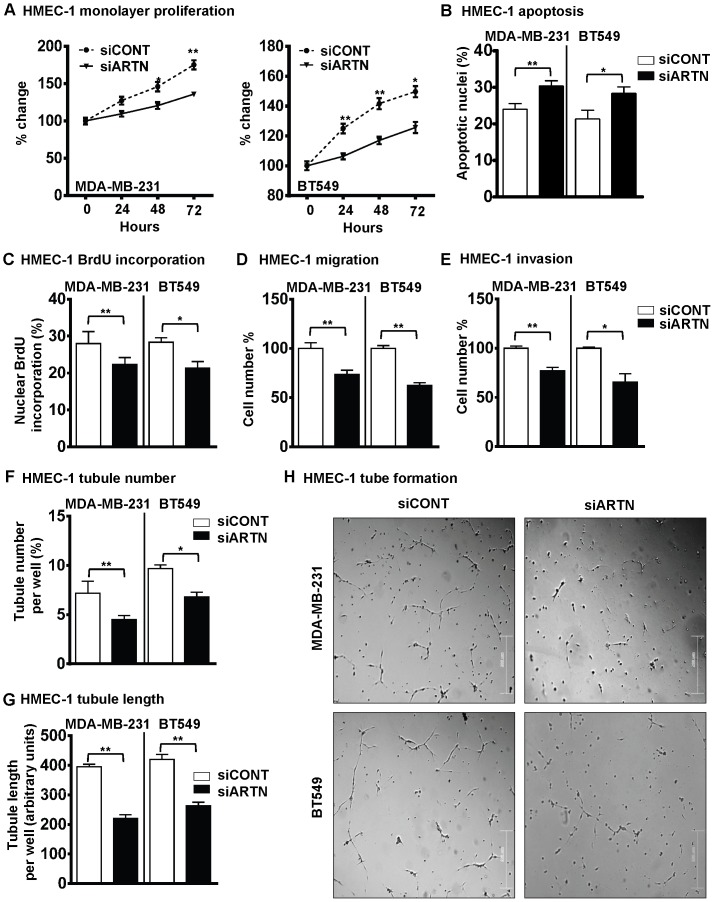
Depleted expression of ARTN in ER-MC cells decreases the angiogenic potential of HMEC-1 cells. (A) HMEC-1 monolayer proliferation. HMEC-1 total cell numbers after indirect co-culture with MDA-MB-231 or BT549 cells with depleted expression of ARTN in 2% serum media. Scrambled siRNA was used as control (siCONT). Cell growth was measured at the indicated time points. (B) HMEC-1 apoptotic cell death after 24 h indirect co-culture with MDA-MB-231 or BT549 cells with depleted expression of ARTN in serum free conditions. (C) HMEC-1 cell cycle progression after 24 h indirect co-culture with MDA-MB-231 or BT549 cells with depleted expression of ARTN in serum free conditions. (D) HMEC-1 cell migration after 24 h indirect co-culture with MDA-MB-231 or BT549 cells with depleted expression of ARTN. (E) HMEC-1 cell invasion assay after 24 h indirect co-culture with MDA-MB-231 or BT549 cells with depletion expression of ARTN. (F) and (G) HMEC-1 cells *in* *vitro* tube formation on matrigel after 12 h indirect co-culture with MDA-MB-231 or BT549 cells with depleted expression of ARTN. HMEC-1 tubule number (F) and tubule length (G) was assessed after 12 h. Tubule number was calculated as (number of cells with tubule/total number of cells counted) × 100, whereas tubule length was calculated as an arbitrary unit using ImageJ software®. (H) Representative light microscopy images of tube formation. Bar 200 µm.*, *p*<0.05; **, *p*<0.01.

We next examined the effects of depletion of endogenous ARTN from ER-MC cells on angiogenic behaviour of HMEC-1 cells. We utilized MDA-MB-231-siARTN and BT549-siARTN cell lines stably transfected with siRNA to ARTN as previously described [8]. Scrambled control siRNA was used to establish the respective siCONT cells [8]. Expression levels of both cellular and secretory ARTN proteins in these respective groups are presented in figure S1A–B. Respective siCONT and siARTN cells were co-cultured with HMEC-1 to determine the effects of depletion of ARTN on monolayer proliferation (total cell count), invasion, migration and tube formation. Depletion of ARTN expression in MDA-MB-231 or BT549 cells decreased HMEC-1 monolayer proliferation by 23% and 16% as compared to respective siCONT control cells (Figure 4A). Next, we examined the effect of depletion of ARTN from ER-MC cells on HMEC-1 cell cycle progression and apoptotic cell death. Indirect co-culture of HMEC-1 cells with MDA-MB-231 or BT549 cells with depleted expression of ARTN significantly increased HMEC-1 apoptotic cell death (29% and 22%) when compared with HMEC-1 cells co-cultured with respective siCONT cells (Figure 4B). Analysis of BrdU incorporation demonstrated that co-culture of HMEC-1 cells with MDA-MB-231 or BT549 cells with depleted expression of ARTN exhibited decreased HMEC-1 cell cycle progression (17% and 26%) when compared with respective siCONT cells co-cultured with HMEC-1 cells (Figure 4C).

Indirect co-culture of MDA-MB-231 or BT549 cells with depletion of ARTN significantly decreased HMEC-1 cell migration (28% and 36%) and cell invasion (23% and 31%) over a 24 hr period when compared with respective siCONT cells (Figure 4D and E). Depletion of ARTN in ER-MC cells also significantly decreased the tubule number (40% and 33%) and tubule length (43% and 35%) generated by HMEC-1 cells compared with HMEC-1 cells co-cultured with the respective MDA-MB-231 or BT549 siCONT cells (Figure 4F–H).

To further verify our previous data we utilized an alternate siRNA to ARTN (siARTN A) [10] to selectively deplete ARTN expression in BT549 cells. Scrambled siRNA was used as control [8]. Depletion of ARTN by siARTN-A in BT549-VEC cells was confirmed by western blot (Figure S2A). As previously observed, siRNA mediated depletion of ARTN with the alternate siRNA to ARTN also resulted in decreased HMEC-1 mediated monolayer proliferation (20%), cell invasion (30%) and 3D matrigel tubule formation (25%) compared to BT549 siCONT cells (Figure S2B–E).

### ARTN Activation of TWIST1 in ER-MC is Upstream of VEGF-mediated Angiogenic Behaviour of HMEC-1 Cells

**Figure 5:**
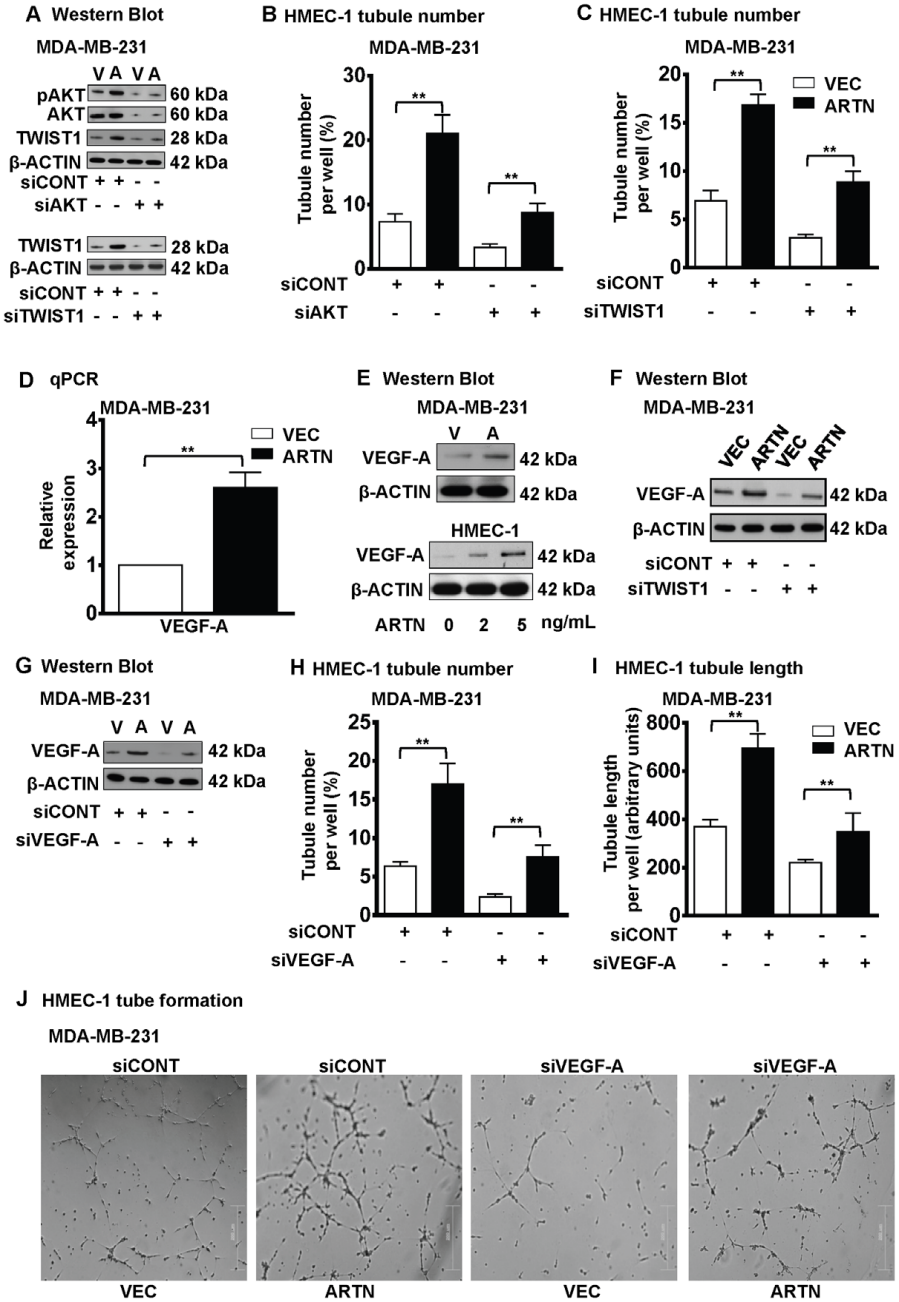
VEGF-A is downstream of ARTN stimulated TWIST1 activation in ER-MC. (A) Western blot analyses for phospho AKT Ser 473 (pAKT), total AKT and TWIST1 expression in MDA-MB-231 cells with forced expression of ARTN± siRNA to AKT (upper panel) or TWIST1 expression in MDA-MB-231 cells with forced expression of ARTN ±siRNA to TWIST1 (lower panel). Scrambled RNA was used as siCONT. β-ACTIN was used as loading control for cell lysates. The sizes of detected protein bands in kiloDalton (kDa) are shown on the right. Here “V” used as MDA-MB-231-VEC cells and “A” used as MDA-MB-231-ARTN cells respectively. (B) HMEC-1 tubule number was assessed after 12 h indirect co-culture with forced expression of ARTN cells of MDA-MB-231± siAKT. Tubule number was calculated as (number of cells with tubule/total number of cells counted) × 100. (C) HMEC-1 tubule number was assessed after 12 h indirect co-culture with forced expression of ARTN cells of MDA-MB-231± TWIST1 siRNA. Tubule number was calculated as (number of cells with tubule/total number of cells counted) × 100. (D) *VEGF-A* mRNA levels were determined by q-PCR in MDA-MB-231 cells with forced expression of ARTN and expressed as the relative expression compared to the MDA-MB-231-VEC cells. (E) Western blot analysis of VEGF-A in MDA-MB-231-VEC and -ARTN cells (upper panel) and for VEGF-A in HMEC-1 cells were assessed in the presence of different concentration of recombinant ARTN (lower panel). β-ACTIN was used as loading control for cell lysates. The sizes of detected protein bands in kDa are shown on the right. Here “V” used as MDA-MB-231-VEC cells and “A” used as MDA-MB-231-ARTN cells respectively. (F) Western blot analyses for VEGF-A in MDA-MB-231-VEC and -ARTN cells ± siRNA to TWIST1. Universal negative control was used as an internal control. β-ACTIN was used as loading control for cell lysates. The sizes of detected protein bands in kDa are shown on the right. (G) Western blot analyses for VEGF-A expression in MDA-MB-231 cells ± siRNA to VEGF-A. Scrambled RNA was used as siCONT. β-ACTIN was used as loading control for cell lysates. The sizes of detected protein bands in kiloDalton (kDa) are shown on the right. Here “V” used as MDA-MB-231-VEC cells and “A” used as MDA-MB-231-ARTN cells respectively. (H) HMEC-1 tubule number and (I) tubule length was assessed after 12 h indirect co-culture with forced expression of ARTN cells of MDA-MB-231± siRNA to VEGF-A. Scrambled RNA was used as siCONT. Tubule number was calculated as (number of cells with tubule/total number of cells counted) × 100, whereas tubule length was calculated as an arbitrary units using ImageJ software®. (J) Representative light microscopy images of tube formation with MDA-MB-231 cells with forced expression of ARTN or control VEC ± siVEGF-A. Bar 200 µm. **, *p*<0.01.

We have previously reported that AKT/TWIST1 mediates ARTN stimulated invasion of ER-MC cells [8]. AKT and TWIST1 have also been implicated to possess a functional role in *de novo* angiogenesis in mammary carcinoma [14], [19]. PI3K/AKT signalling is an important pathway regulating various pro-angiogenic factors, such as HIF1α and VEGF-A [20]. To determine if AKT mediates ARTN enhancement of the angiogenic potential of ER-MC cells, we first employed siRNA to AKT1 to deplete AKT and therefore inhibit AKT activity in MDA-MB-231 cells. Treatment of MDA-MB-231-VEC cells with siAKT decreased the basal levels of pAKT and total AKT. ARTN stimulated activation of AKT was also decreased by siAKT (Figure 5A, upper panel). siAKT also decreased the basal level of TWIST1 expression in MDA-MB-231-VEC cells and also significantly inhibited ARTN stimulated TWIST1 expression in MDA-MB-231-ARTN cells, as previously demonstrated using AKT inhibitor IV [8]. Depletion of AKT in MDA-MB-231 cells abrogated the stimulatory effects of ER-MC cells on HMEC-1 tube formation (Figure 5B). Furthermore, treatment with AKT inhibitor IV in MDA-MB-231 cells with forced expression of ARTN [8] also resulted in decreased HMEC-1 tube formation (Figure S3).

We have previously demonstrated [8] that AKT activation by ARTN in MDA-MB-231 cells resulted in increased expression of TWIST1. Forced expression of TWIST1 in ER-MC cells has been reported to produce increased tumor angiogenesis resulting in an aggressive carcinoma phenotype [14]. To determine whether TWIST1 mediates ARTN stimulation of the angiogenic potential of ER-MC cells, we employed siRNA to TWIST1 [8] to selectively deplete TWIST1 expression in MDA-MB-231 cells. siTWIST1 selectively reduced TWIST1 protein expression in MDA-MB-231-VEC cells as evident in Figure 5A, lower panel. β-ACTIN was used an internal control. Depletion of TWIST1 abrogated the stimulatory effects of ARTN from both MDA-MB-231-VEC and cells with forced expression of ARTN on HMEC-1 tube formation (Figure 5C).

It has been previously reported that VEGF-A mediates at least some of the angiogenic effects of mammary carcinoma cells [17]. q-PCR gene expression data demonstrated that forced expression of ARTN in MDA-MB-231 cells significantly increased *VEGF-A* mRNA expression as compared to control cells (Figure 5D). Similarly, forced expression of ARTN in MDA-MB-231 cells increased VEGF-A protein expression compared to control cells (Figure 5E upper panel). Furthermore, we demonstrated that exogenously added ARTN increased VEGF-A expression in HMEC-1 cells. Higher concentrations of exogenously added ARTN resulted in increased VEGF-A expression in HMEC-1 cells (Figure 5E lower panel).

TWIST1 signalling has been reported to promote VEGF-A expression in mammary carcinoma cells [14] and we have previously demonstrated that ARTN increased TWIST1 expression to modulate ARTN stimulated oncogenicity [8]. We therefore next determined if depletion of TWIST1 by siRNA would abrogate ARTN dependent VEGF-A expression in MDA-MB-231 cells with forced expression of ARTN. As observed [8], western blot analysis demonstrated that depletion of TWIST1 in MDA-MB-231-VEC cells reduced the basal level of VEGF-A expression compared to MDA-MB-231-VEC cells with siCONT. Further, depletion of TWIST1 by siRNA also specifically abrogated the enhanced VEGF-A expression observed in MDA-MB-231-ARTN cells as compared to MDA-MB-231-ARTN cells with siCONT (Figure 5F).

We next analysed the depletion of VEGF-A expression in ER-MC cells on the ability of ARTN to stimulate tube formation by HMEC-1 cells. We employed siRNA to VEGF-A to selectively reduce VEGF-A expression in MDA-MB-231 cells. siVEGF-A selectively reduced VEGF-A protein expression in MDA-MB-231-VEC and MDA-MB-231-ARTN cells as observed in Figure 5G. Depletion of VEGF-A in MDA-MB-231-VEC cells reduced tube formation by HMEC-1 cells in co-culture compared to co-culture with MDA-MB-231-VEC cells transfected with siCONT (Figure 5H). Depletion of VEGF-A in MDA-MB-231-ARTN cells also partially abrogated the stimulatory effects of ARTN on HMEC-1 tubule number and tubule length (Figure 5H–J). We also analysed the functional effects on HMEC-1 cells of VEGF-A antagonism in MDA-MB-231 cells with forced expression of ARTN. Functional antagonism of VEGF-A was achieved using the inhibitory humanized monoclonal antibody bevacizumab [21]. Functional antagonism of VEGF-A with bevacizumab partially abrogated ARTN-mediated stimulation of HMEC-1 tube formation when co-cultured with MDA-MB-231 cells with forced expression of ARTN (Figure S4A–B). Bevacizumab also significantly reduced HMEC-1 tube formation when co-cultured with MDA-MB-231-VEC cells as expected [17] (Figure S4A–B).

We lastly investigated the effects of combined functional antagonism of ARTN and VEGF-A from ER-MC cells (MDA-MB-231) on the ability of HMEC-1 cells to form tubules. Treatment of MDA-MB-231-WT cells with bevacizumab alone significantly decreased tubule number (Figure S4C). Combined depletion of ARTN with siRNA to ARTN and treatment with bevacizumab resulted in a further reduction in tubule number (Figure S4C). ARTN therefore possesses both VEGF-A dependent and VEGF-A independent effects on endothelial cell behaviour.

### Correlation between ARTN and VEGF-A Expression in ER-MC

We next determined a potential correlation between VEGF-A expression and ARTN expression in a cohort of patients with ER-MC by IHC analysis. IHC analysis showed that ARTN and VEGF-A protein were highly expressed in ER-MC (Figure S5). To assess any possible correlation between ARTN and VEGF expression, we stratified the expression level of each protein into two categories (low and high expression). Of those tumors with low expression of ARTN, only 17.1% exhibited high VEGF expression whereas 56.6% of tumors with high ARTN expression also exhibited high VEGF expression. To further confirm the correlation of expression between ARTN and VEGF, we compared the relationship by Spearman’s rank correlation co-efficient and found a significant positive value (Spearman correlation: rs = 0.301, *P* = 0.008) (Table 1). Thus, expression of ARTN in ER-MC partially correlates with VEGF-A expression.

**Table 1:** Correlation between ARTN and VEGF-A expression in ER negative mammary carcinoma.

		ARTN expression	
		Low	High
VEGF expression	Low	11 (14.5%)	9 (11.8%)
	High	13 (17.1%)	43 (56.6%)

**Spearman correlation:** *P*  = 0.008 r_s_  = 0.301.

**Footnotes:**

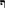
Expression parameters are described in details in the *Materials and methods* section.

### ARTN Secreted from ER-MC Cells Promotes Tumor Angiogenesis *in vivo*

**Figure 6:**
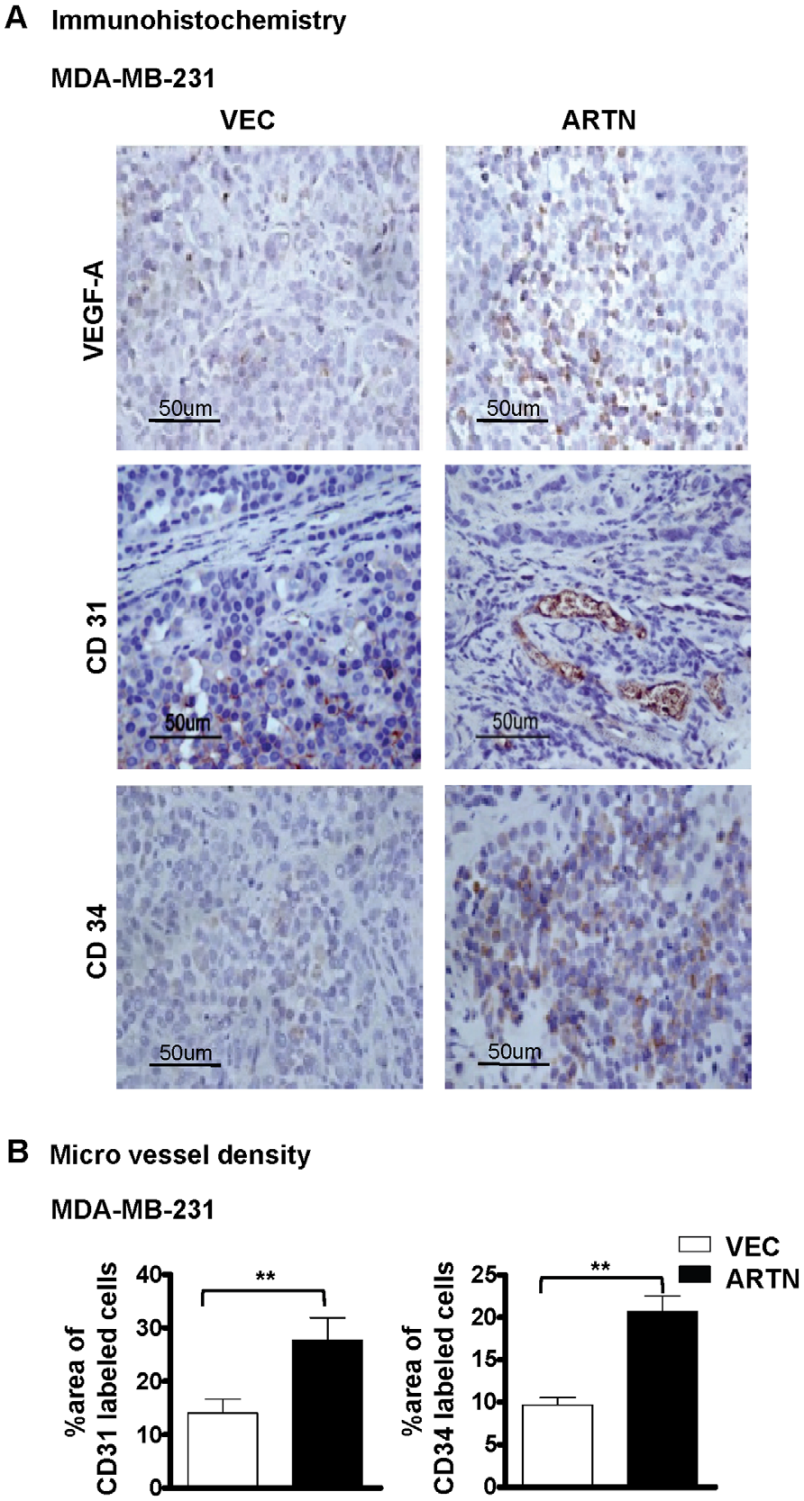
Forced expression of ARTN in ER-MC cells stimulates tumor angiogenesis. (A) Immunohistochemical analysis of VEGF-A, CD31 and CD34 protein levels in xenograft tumors formed by MDA-MB-231 cells with forced expression of ARTN and control VEC. (B) Microvessel density (MVD) was assessed by quantifying percentage of area of CD31 or CD34 labelled cells in xenograft formed by either MDA-MB-231 cells with forced expression of ARTN or control VEC.

To determine the potential *in vivo* role of ARTN secreted from ER-MC cells in tumor angiogenesis, we injected MDA-MB-231-ARTN cells into the mammary fat pad of immunodeficient nude mice. After 4 weeks, MDA-MB-231-ARTN tumors grew significantly larger than MDA-MB-231-VEC tumors as described [8]. Previous reports indicate that higher expression of VEGF-A correlates with increased tumor vasculature in patients with invasive mammary carcinoma [22]. We therefore determined the expression of VEGF-A by IHC in xenograft tumors derived from MDA-MB-231-VEC and MDA-MB-231-ARTN cells. VEGF-A protein expression was higher in xenografts derived from MDA-MB-231-ARTN cells as compared to xenografts derived from control VEC cells (Figure 6A).

To examine microvessel density (MVD), CD31 and CD34 protein expression was also determined by immunohistochemistry. We observed an increased area of CD31 and CD34-labelled cells in xenografts derived from MDA-MB-231-ARTN as compared to xenograft derived from MDA-MB-231-VEC tumors, indicating significantly increased tumor microvessel density (Figure 6B). Thus expression of ARTN in ER-MC cells promotes tumor angiogenesis *in vivo*.

## Discussion

Tumor angiogenesis is proposed to be multistep process that permits tumor cells to access the circulation and metastasize to distant organ sites [23]. Undoubtedly, VEGF-A is an important mediator of angiogenesis in mammary carcinoma. VEGF-A is reported to be predominantly expressed in early stage mammary carcinoma [24]. However, during malignant progression several other known molecular mediators may also interplay to promote angiogenesis [25], [26]. Therefore targeting VEGF-A alone would not be sufficient to provide maximal potential therapeutic benefits. Herein we have demonstrated that ARTN directly regulates endothelial cell function. Furthermore, we have demonstrated that ARTN secreted by ER-MC cells also regulates endothelial cell function, at least in part by increasing the expression of VEGF-A from mammary carcinoma cells. ARTN may also, in addition to VEGF-A, regulate other factors promoting angiogenesis. Indeed, gene expression studies on MCF-7 cells with forced expression of ARTN [4] demonstrated that ARTN regulates several pro-angiogenic factors, including *MMP1* [27] and *PLAU* [28]. We have also observed increased *IL-8* expression in BT549 cells with forced expression of ARTN (unpublished observations). IL-8 is a well described promoter of tumor angiogenesis [29]. Thus, apart from direct actions on endothelial cells, ARTN also co-ordinately regulates a pro-angiogenic programme of gene expression from mammary carcinoma cells. Recently, cancer stem cells (CSCs) have been reported to secrete angiogenic factors such as VEGF-A to provide vasculature to support CSC renewal [30]. In this regard, it is interesting that we have reported that ARTN promotes CSC-like behaviour in ER-MC cell lines (manuscript submitted). Thus, ARTN modulation of endothelial cell behaviour promoting *de novo* angiogenesis may occur as part of a co-ordinated tumor growth process involving cells with CSC-like behaviour. Indeed, similar to ARTN, VEGF-A has been reported to promote cancer cell proliferation and metastasis [31], tumor growth [32], CSC-like behaviour [33], and angiogenesis [34]. VEGF-A is apparently one mediator of the co-ordinated oncogenic properties of ARTN [4], [8] which now includes angiogenesis.

We report herein that ARTN regulation of VEGF-A is mediated by AKT-TWIST1. Concordant with our results herein and previously [8], higher expression of AKT, TWIST1 and VEGF-A has been reported in metastatic mammary carcinoma [12], [14], [16]. ARTN has also been reported to utilize the AKT-TWIST1 pathway for stimulation of CSC-like behaviour (manuscript submitted). Given that VEGF-A also stimulates CSC-like behaviour it is plausible that VEGF-A partially mediates the CSC-like promoting behaviours of ARTN. Interestingly, ARTN also promotes the expression of other secreted proteins, such as TFF3 [10] which also promotes the oncogenic behaviour of mammary carcinoma cells [35] and possesses angiogenic activity [36]. ARTN may therefore stimulate a co-ordinated programme of gene expression promoting oncogenicity of mammary carcinoma and progression of the clinical disease.

ARTN, like other GFLs has been reported to utilize GFRα mediated RET signalling [18]. We have demonstrated herein that ARTN regulates VEGF-A in ER-MC cells. Interestingly, MDA-MB-231 cells do not express RET [37], yet ARTN activates the AKT pathway in MDA-MB-231 cells [8]. It is relevant that GFLs, including ARTN, bind to and/or activate multiple receptors/signalling pathways [18]. Indeed, ARTN has recently been demonstrated to bind and activate syndecan-3, leading to activation of Src kinase, in addition to binding to GFRα3 and GFRα1 [38]. It is possible that different upstream signalling pathways converge to activate AKT leading to increased VEGF-A expression via TWIST1 and ARTN may therefore activate the AKT-TWIST1-VEGF-A axis independent of RET. The precise upstream signalling pathways utilized by ARTN to regulate VEGF-A remain to be determined. ARTN may also utilize other signalling pathways to regulate distinct alternate pro-angiogenic genes.

In summary, we have demonstrated herein that ARTN promotes *de novo* tumor angiogenesis mediated in part by increased VEGF-A expression. ARTN therefore co-ordinately regulates processes promoting tumor growth and metastasis. Such co-ordinate regulation of tumor growth and metastasis warrants consideration of the use of therapeutic strategies inhibitory to ARTN activity in ER-MC.

## Materials and Methods

### Cell Culture

Cell lines used in this study were obtained from the ATCC (American Type Culture Collection) and cultured as recommended. Generation of MDA-MB-231 or BT549 cells with forced expression or depleted expression of ARTN have been previously described [8]. Human microvascular endothelial cells (HMEC-1) were cultured as described previously [17].

### Reagents

Recombinant human ARTN was purchased from Peprotech inc, NJ, USA. AKT Inhibitor IV and siRNA to TWIST1 were purchased as previously described [8]. Stealth siRNAs to AKT, TWIST1 and VEGF-A were purchased from Invitrogen Inc, CA. Bevacizumab was purchased from Roche Diagnostics, NZ. siRNA to ARTN (siARTN-A) were as previously described [10].

### Polymerase Chain Reaction

Quantitative real time PCR primers were used as described earlier [6]. q-PCR was performed as described previously [8] and VEGF-A primers used were identical to those in a previous report [17].

### Immunoblotting

Western blot analysis was performed as described earlier [8] using the following antibodies: goat anti-ARTN polyclonal antibody (R&D Systems, Minneapolis, MN) mouse anti-β-ACTIN monoclonal antibody (Sigma, St Louis, MO) and mouse anti-VEGF-A monoclonal antibody (SantaCruz, CA). pAKT(Ser473), total AKT and TWIST1 antibody were used as previously mentioned [8].

### Cell Function Assays

Indirect co-culture of mammary carcinoma cells with HMEC-1 cells for determination of monolayer proliferation assay, cell migration, cell invasion, tube formation assays were performed as previously described [17]. Monolayer proliferation assay, cell migration and cell invasion assay were performed as described previously [8] and tubule formation assay was performed as stated earlier [17]. Apoptosis assay was performed as described previously [9] using Annexin-V-FLUOS staining kit (Roche, Mannheim, Germany) and BrdU assays were performed as described previously [17].

### Tumor Xenograft in Nude Mice

All animal work was done in accordance with a protocol approved by the institutional animal care and use committee. Tumor growth was achieved as mentioned earlier [8]. Immunohistochemical (IHC) analysis of paraffin-embedded specimens was performed as described previously [4]. In short, 6 µm tissue sections were cut, sections were deparaffinised in xylene, rehydrated in a graded series of ethanol solutions, and heated in a microwave oven in 0.01 M sodium citrate buffer (pH 6.0) for 10 minutes for antigen retrieval. All of the antibodies including anti-VEGF (Cat No.MAB-0243, 1∶50), anti-CD31 (Cat No.MAB-0031, 1∶50) and anti-CD34 (Cat No.MAB-0034, 1∶50) were bought from Maixin Biotechnology Development Co.Ltd (Fuzhou, China).

### Histopathological Analysis

Tissue samples were collected from 76 female breast cancer patients with ER-MC from the First Affiliated Hospital of Anhui Medical University (Hefei, P. R. China) presenting between 2001 and 2002. Institutional ethics committee approval for the project was obtained before commencement of the study and was in compliance with the Helsinki Declaration. Immunohistochemical analysis of paraffin-embedded specimens was performed as described previously [4].

### Statistics

All numerical data are expressed as mean±S.E.M. and statistical significance was assessed by Student’s *t*-test (P<0.05 was considered as significant) using Microsoft Excel XP unless otherwise indicated (χ2 test).

## Supporting Information

Figure S1 Western blot analysis for ARTN in (A) wild type cells of MDA-MB-231 and BT549 or (B) in MDA-MB-231 and BT549 cells with forced expression or depletion of ARTN. β-ACTIN was used as loading control for cell lysates. The sizes of detected protein bands in kiloDalton (kDa) are shown on the right. Soluble whole cellular extracts or concentrated conditioned media were run on an SDS-PAGE.
(TIF)

Figure S2 Depleted expression of ARTN decreases angiogenic potential of HMEC-1 cells. (A) Western blot analyses for ARTN in BT549 cells with siRNA mediated depletion of ARTN. β-ACTIN was used as loading control for cell lysates. The sizes of detected protein bands in kiloDalton (kDa) are shown on the right. (B) HMEC-1 monolayer proliferation. HMEC-1 total cell numbers after indirect co-culture with BT549 cells with depleted expression of ARTN in 2% serum media. Cell growth was measured at the indicated time points. Scrambled siRNA was used as control (siCONT). (C) HMEC-1 cell invasion assay after 24 h indirect co-culture with BT549 cells with depletion expression of ARTN. (D) and (E) HMEC-1 cells *in* *vitro* tube formation on matrigel after 12 h indirect co-culture with BT549 cells with depleted expression of ARTN. HMEC-1 tube formation was assessed after 12 h. Tubule number was calculated as (number of cells with tubule/total number of cells counted) × 100. Bar 200 µm.*, *p*<0.05; **, *p*<0.01.
(TIF)

Figure S3 HMEC-1 tubule number was assessed after 12 h indirect co-culture with forced expression of ARTN cells of MDA-MB-231± AKT inhibitor IV as previously described [8]. Tubule number was calculated as (number of cells with tubule/total number of cells counted) × 100. **, *p*<0.01.
(TIF)

Figure S4 VEGF-A is downstream of ARTN stimulated angiogenesis in ER-MC. (A) HMEC-1 tubule number and (B) tubule length was assessed after 12 h indirect co-culture with forced expression of ARTN cells of MDA-MB-231± bevacizumab. Human IgG was used as control. Tubule number was calculated as (number of cells with tubule/total number of cells counted) × 100, whereas tubule length was calculated as an arbitrary units using ImageJ software®. (C) HMEC-1 tubule number was assessed after 12 h indirect co-culture with MDA-MB-231-wild type (MDA-MB-231-WT) in the presence of either bevacizumab (0.5 mg/mL) alone or with siRNA to ARTN. Control cells were treated with human IgG, siCONT of ARTN. Tubule number was calculated as (number of cells with tubule/total number of cells counted) × 100. *, *p*<0.05; **, *p*<0.01.
(TIF)

Figure S5 Immunohistochemistry (IHC) images of ARTN and VEGF-A expression in ER-MC. (A) ARTN and VEGF-A expression was detected by IHC analysis in ER-MC samples. Both ARTN and VEGF-A are predominantly localized to cytoplasm of carcinoma cells. X200 magnifications.
(TIF)

## References

[1] Schneider BP, Miller KD (2005) Angiogenesis of Breast Cancer. Journal of Clinical Oncology 23: 1782–1790. 15755986 10.1200/JCO.2005.12.017

[2] Loges S, Mazzone M, Hohensinner P, Carmeliet P (2009) Silencing or fueling metastasis with VEGF inhibitors: antiangiogenesis revisited. Cancer Cell 15: 167–170. 19249675 10.1016/j.ccr.2009.02.007

[3] Paez-Ribes M, Allen E, Hudock J, Takeda T, Okuyama H, et al (2009) Antiangiogenic therapy elicits malignant progression of tumors to increased local invasion and distant metastasis. Cancer Cell 15: 220–231. 19249680 10.1016/j.ccr.2009.01.027 PMC2874829

[4] Kang J, Perry JK, Pandey V, Fielder GC, Mei B, et al (2009) Artemin is oncogenic for human mammary carcinoma cells. Oncogene 28: 2034–2045. 19363524 10.1038/onc.2009.66

[5] Ceyhan GO, Giese NA, Erkan M, Kerscher AG, Wente MN, et al (2006) The neurotrophic factor artemin promotes pancreatic cancer invasion. Ann Surg 244: 274–281. 16858191 10.1097/01.sla.0000217642.68697.55 PMC1602177

[6] Pandey V, Qian PX, Kang J, Perry JK, Mitchell MD, et al (2010) Artemin stimulates oncogenicity and invasiveness of human endometrial carcinoma cells. Endocrinology 151: 909–920. 20118197 10.1210/en.2009-0979

[7] Tang J-Z, Kong X-J, Kang J, Fielder GC, Steiner M, et al (2010) Artemin-Stimulated Progression of Human Non Small Cell Lung Carcinoma Is Mediated by BCL2. Molecular Cancer Therapeutics 9: 1697–1708. 20530713 10.1158/1535-7163.MCT-09-1077

[8] Banerjee A, Wu ZS, Qian P, Kang J, Pandey V, et al (2011) ARTEMIN synergizes with TWIST1 to promote metastasis and poor survival outcome in patients with ER negative mammary carcinoma. Breast Cancer Res 13: R112. 22060274 10.1186/bcr3054 PMC3326554

[9] Pandey V, Jung Y, Kang J, Steiner M, Qian PX, et al (2010) Artemin Reduces Sensitivity to Doxorubicin and Paclitaxel in Endometrial Carcinoma Cells through Specific Regulation of CD24. Transl Oncol 3: 218–229. 20689763 10.1593/tlo.09325 PMC2915413

[10] Kang J, Qian PX, Pandey V, Perry JK, Miller LD, et al (2010) Artemin is estrogen regulated and mediates antiestrogen resistance in mammary carcinoma. Oncogene 29: 3228–3240. 20305694 10.1038/onc.2010.71

[11] Romon R, Adriaenssens E, Lagadec C, Germain E, Hondermarck H, et al (2010) Nerve growth factor promotes breast cancer angiogenesis by activating multiple pathways. Mol Cancer 9: 157. 20569463 10.1186/1476-4598-9-157 PMC2901260

[12] Tsutsui S, Matsuyama A, Yamamoto M, Takeuchi H, Oshiro Y, et al (2010) The Akt expression correlates with the VEGF-A and -C expression as well as the microvessel and lymphatic vessel density in breast cancer. Oncol Rep 23: 621–630. 20126999 10.3892/or_00000677

[13] Jiang BH, Liu LZ (2008) PI3K/PTEN signaling in tumorigenesis and angiogenesis. Biochim Biophys Acta 1784: 150–158. 17964232 10.1016/j.bbapap.2007.09.008

[14] Mironchik Y, Winnard PT Jr, Vesuna F, Kato Y, Wildes F, et al (2005) Twist overexpression induces in vivo angiogenesis and correlates with chromosomal instability in breast cancer. Cancer Res 65: 10801–10809. 16322226 10.1158/0008-5472.CAN-05-0712 PMC5575828

[15] Hu L, Roth JM, Brooks P, Ibrahim S, Karpatkin S (2008) Twist is required for thrombin-induced tumor angiogenesis and growth. Cancer Res 68: 4296–4302. 18519689 10.1158/0008-5472.CAN-08-0067

[16] Kallergi G, Papadaki MA, Politaki E, Mavroudis D, Georgoulias V, et al (2011) Epithelial to mesenchymal transition markers expressed in circulating tumour cells of early and metastatic breast cancer patients. Breast Cancer Res 13: R59. 21663619 10.1186/bcr2896 PMC3218948

[17] Brunet-Dunand SE, Vouyovitch C, Araneda S, Pandey V, Vidal LJ, et al (2009) Autocrine human growth hormone promotes tumor angiogenesis in mammary carcinoma. Endocrinology 150: 1341–1352. 18974274 10.1210/en.2008-0608

[18] Airaksinen MS, Saarma M (2002) The GDNF family: signalling, biological functions and therapeutic value. Nat Rev Neurosci 3: 383–394. 11988777 10.1038/nrn812

[19] Zheng X, Jiang F, Katakowski M, Zhang ZG, Lu QE, et al (2009) ADAM17 promotes breast cancer cell malignant phenotype through EGFR-PI3K-AKT activation. Cancer Biol Ther 8: 1045–1054. 19395875 10.4161/cbt.8.11.8539 PMC2766867

[20] Skinner HD, Zheng JZ, Fang J, Agani F, Jiang BH (2004) Vascular endothelial growth factor transcriptional activation is mediated by hypoxia-inducible factor 1alpha, HDM2, and p70S6K1 in response to phosphatidylinositol 3-kinase/AKT signaling. J Biol Chem 279: 45643–45651. 15337760 10.1074/jbc.M404097200

[21] Loges S, Schmidt T, Carmeliet P (2010) Mechanisms of resistance to anti-angiogenic therapy and development of third-generation anti-angiogenic drug candidates. Genes Cancer 1: 12–25. 21779425 10.1177/1947601909356574 PMC3092176

[22] El Agouza IM, Eissa SS, El Houseini MM, El-Nashar DE, Abd El Hameed OM (2011) Taurine: a novel tumor marker for enhanced detection of breast cancer among female patients. Angiogenesis 14: 321–330. 21553281 10.1007/s10456-011-9215-3

[23] Giovannini M, Aldrighetti D, Zucchinelli P, Belli C, Villa E (2010) Antiangiogenic strategies in breast cancer management. Crit Rev Oncol Hematol 76: 13–35. 20702105 10.1016/j.critrevonc.2009.12.004

[24] Relf M, LeJeune S, Scott PA, Fox S, Smith K, et al (1997) Expression of the angiogenic factors vascular endothelial cell growth factor, acidic and basic fibroblast growth factor, tumor growth factor beta-1, platelet-derived endothelial cell growth factor, placenta growth factor, and pleiotrophin in human primary breast cancer and its relation to angiogenesis. Cancer Res 57: 963–969. 9041202

[25] Cao Y, Liu Q (2007) Therapeutic targets of multiple angiogenic factors for the treatment of cancer and metastasis. Adv Cancer Res 97: 203–224. 17419947 10.1016/S0065-230X(06)97009-2

[26] Shibuya M (2008) Vascular endothelial growth factor-dependent and -independent regulation of angiogenesis. BMB Rep 41: 278–286. 18452647 10.5483/bmbrep.2008.41.4.278

[27] Eck SM, Hoopes PJ, Petrella BL, Coon CI, Brinckerhoff CE (2009) Matrix metalloproteinase-1 promotes breast cancer angiogenesis and osteolysis in a novel in vivo model. Breast Cancer Res Treat 116: 79–90. 18597171 10.1007/s10549-008-0085-3 PMC3772530

[28] Bajou K, Lewalle JM, Martinez CR, Soria C, Lu H, et al (2002) Human breast adenocarcinoma cell lines promote angiogenesis by providing cells with uPA-PAI-1 and by enhancing their expression. Int J Cancer 100: 501–506. 12124797 10.1002/ijc.10487

[29] Lin Y, Huang R, Chen L, Li S, Shi Q, et al (2004) Identification of interleukin-8 as estrogen receptor-regulated factor involved in breast cancer invasion and angiogenesis by protein arrays. Int J Cancer 109: 507–515. 14991571 10.1002/ijc.11724

[30] Zhao Y, Bao Q, Renner A, Camaj P, Eichhorn M, et al (2011) Cancer stem cells and angiogenesis. Int J Dev Biol 55: 477–482. 21732274 10.1387/ijdb.103225yz

[31] Toi M, Matsumoto T, Bando H (2001) Vascular endothelial growth factor: its prognostic, predictive, and therapeutic implications. Lancet Oncol 2: 667–673. 11902537 10.1016/S1470-2045(01)00556-3

[32] Hirakawa S, Kodama S, Kunstfeld R, Kajiya K, Brown LF, et al (2005) VEGF-A induces tumor and sentinel lymph node lymphangiogenesis and promotes lymphatic metastasis. J Exp Med 201: 1089–1099. 15809353 10.1084/jem.20041896 PMC2213132

[33] Beck B, Driessens G, Goossens S, Youssef KK, Kuchnio A, et al (2011) A vascular niche and a VEGF-Nrp1 loop regulate the initiation and stemness of skin tumours. Nature 478: 399–403. 22012397 10.1038/nature10525

[34] Manders P, Beex LV, Tjan-Heijnen VC, Geurts-Moespot J, Van Tienoven TH, et al (2002) The prognostic value of vascular endothelial growth factor in 574 node-negative breast cancer patients who did not receive adjuvant systemic therapy. Br J Cancer 87: 772–778. 12232762 10.1038/sj.bjc.6600555 PMC2364266

[35] Kannan N, Kang J, Kong X, Tang J, Perry JK, et al (2010) Trefoil factor 3 is oncogenic and mediates anti-estrogen resistance in human mammary carcinoma. Neoplasia 12: 1041–1053. 21170268 10.1593/neo.10916 PMC3003139

[36] Ahmed AR, Griffiths AB, Tilby MT, Westley BR, May FE (2012) TFF3 is a normal breast epithelial protein and is associated with differentiated phenotype in early breast cancer but predisposes to invasion and metastasis in advanced disease. Am J Pathol 180: 904–916. 22341453 10.1016/j.ajpath.2011.11.022

[37] Boulay A, Breuleux M, Stephan C, Fux C, Brisken C, et al (2008) The Ret receptor tyrosine kinase pathway functionally interacts with the ERalpha pathway in breast cancer. Cancer Res 68: 3743–3751. 18483257 10.1158/0008-5472.CAN-07-5100

[38] Bespalov MM, Sidorova YA, Tumova S, Ahonen-Bishopp A, Magalhaes AC, et al (2011) Heparan sulfate proteoglycan syndecan-3 is a novel receptor for GDNF, neurturin, and artemin. J Cell Biol 192: 153–169. 21200028 10.1083/jcb.201009136 PMC3019558

