# Supplementary figures and images for: ARTEMIN Promotes De Novo Angiogenesis in ER Negative Mammary Carcinoma through Activation of TWIST1-VEGF-A Signalling
Source: PLoS One. 2012 Nov 21;7(11):e50098. doi: 10.1371/journal.pone.0050098 (PMC3503764; doi:10.1371/journal.pone.0050098)

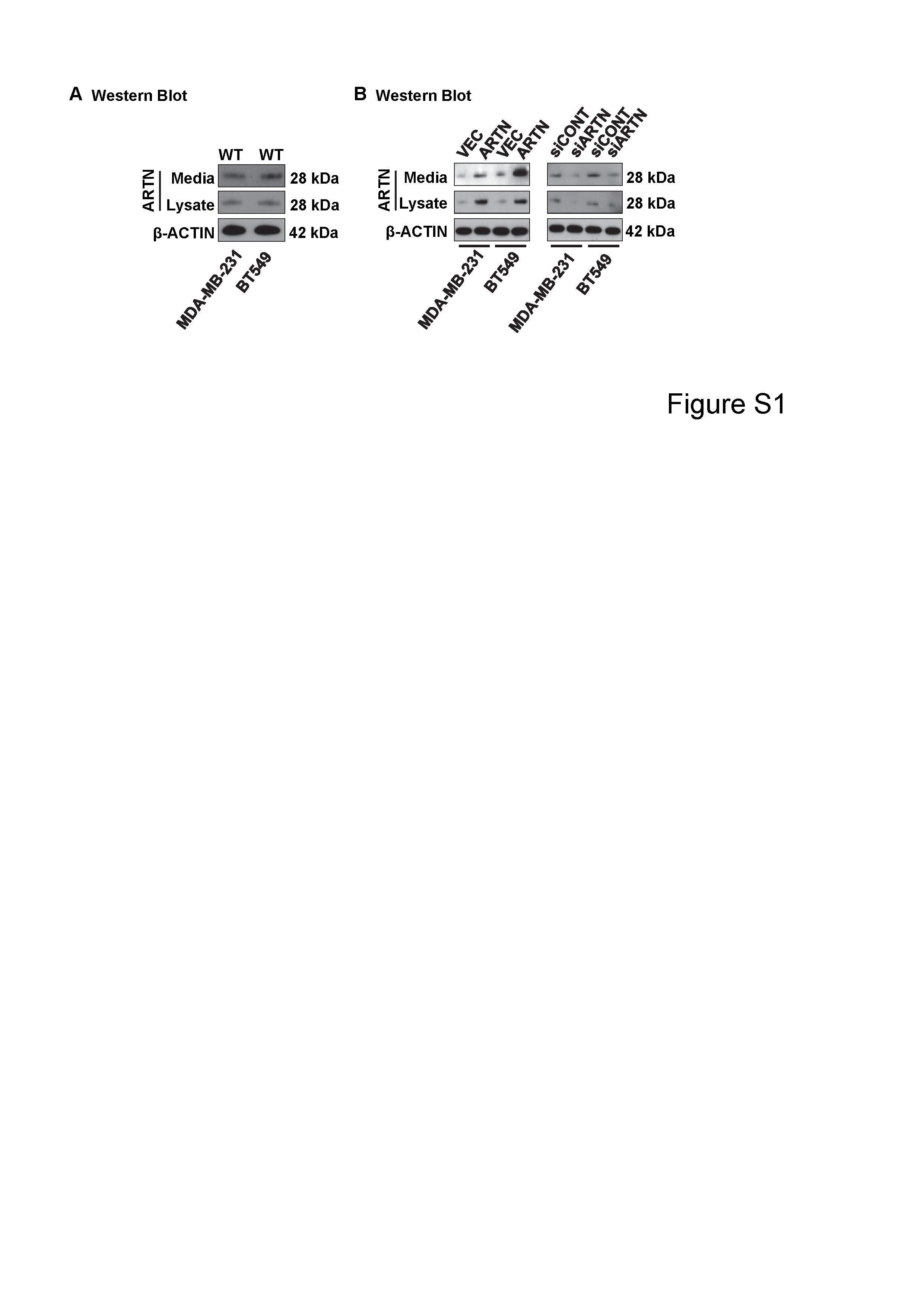

Supplement: Figure S1 — Western blot analysis for ARTN in (A) wild type cells of MDA-MB-231 and BT549 or (B) in MDA-MB-231 and BT549 cells with forced expression or depletion of ARTN. β-ACTIN was used as loading control for cell lysates. The sizes of detected protein bands in kiloDalton (kDa) are shown on the right. Soluble whole cellular extracts or concentrated conditioned media were run on an SDS-PAGE. (TIF) [file pone.0050098.s001.tif]

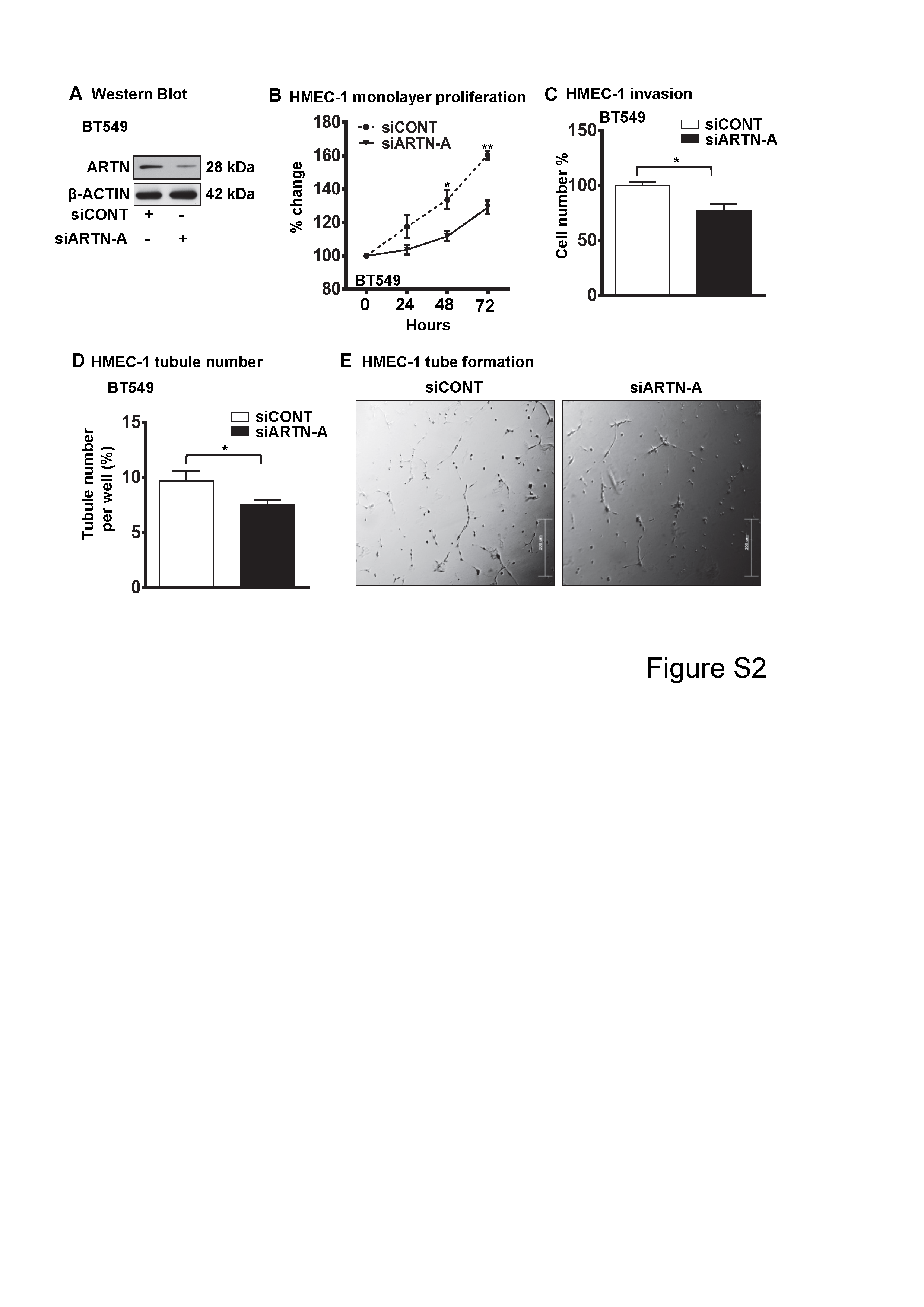

Supplement: Figure S2 — Depleted expression of ARTN decreases angiogenic potential of HMEC-1 cells. (A) Western blot analyses for ARTN in BT549 cells with siRNA mediated depletion of ARTN. β-ACTIN was used as loading control for cell lysates. The sizes of detected protein bands in kiloDalton (kDa) are shown on the right. (B) HMEC-1 monolayer proliferation. HMEC-1 total cell numbers after indirect co-culture with BT549 cells with depleted expression of ARTN in 2% serum media. Cell growth was measured at the indicated time points. Scrambled siRNA was used as control (siCONT). (C) HMEC-1 cell invasion assay after 24 h indirect co-culture with BT549 cells with depletion expression of ARTN. (D) and (E) HMEC-1 cells in vitro tube formation on matrigel after 12 h indirect co-culture with BT549 cells with depleted expression of ARTN. HMEC-1 tube formation was assessed after 12 h. Tubule number was calculated as (number of cells with tubule/total number of cells counted) × 100. Bar 200 µm.*, p<0.05; **, p<0.01. (TIF) [file pone.0050098.s002.tif]

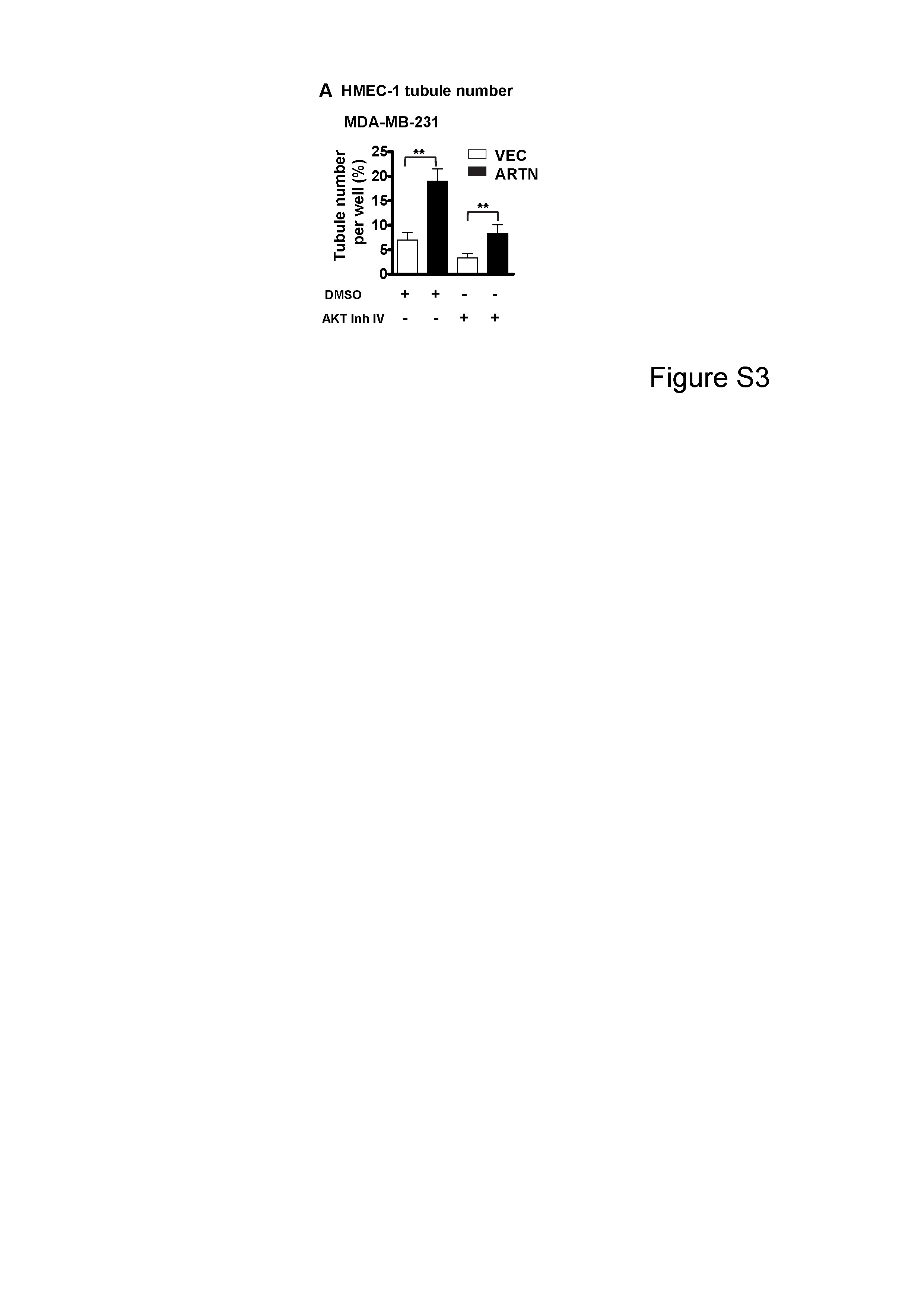

Supplement: Figure S3 — HMEC-1 tubule number was assessed after 12 h indirect co-culture with forced expression of ARTN cells of MDA-MB-231± AKT inhibitor IV as previously described [8]. Tubule number was calculated as (number of cells with tubule/total number of cells counted) × 100. **, p<0.01. (TIF) [file pone.0050098.s003.tif]

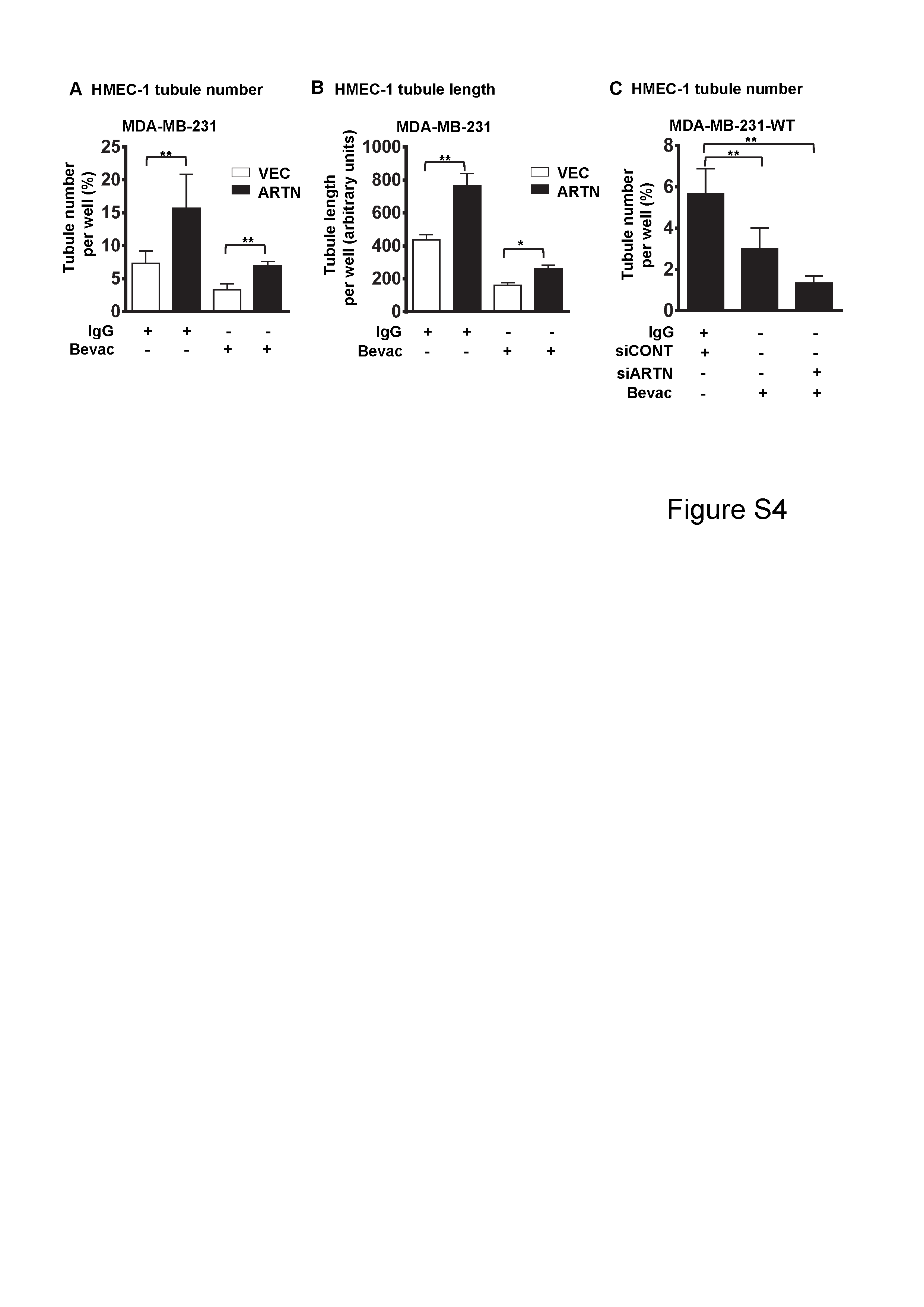

Supplement: Figure S4 — VEGF-A is downstream of ARTN stimulated angiogenesis in ER-MC. (A) HMEC-1 tubule number and (B) tubule length was assessed after 12 h indirect co-culture with forced expression of ARTN cells of MDA-MB-231± bevacizumab. Human IgG was used as control. Tubule number was calculated as (number of cells with tubule/total number of cells counted) × 100, whereas tubule length was calculated as an arbitrary units using ImageJ software®. (C) HMEC-1 tubule number was assessed after 12 h indirect co-culture with MDA-MB-231-wild type (MDA-MB-231-WT) in the presence of either bevacizumab (0.5 mg/mL) alone or with siRNA to ARTN. Control cells were treated with human IgG, siCONT of ARTN. Tubule number was calculated as (number of cells with tubule/total number of cells counted) × 100. *, p<0.05; **, p<0.01. (TIF) [file pone.0050098.s004.tif]

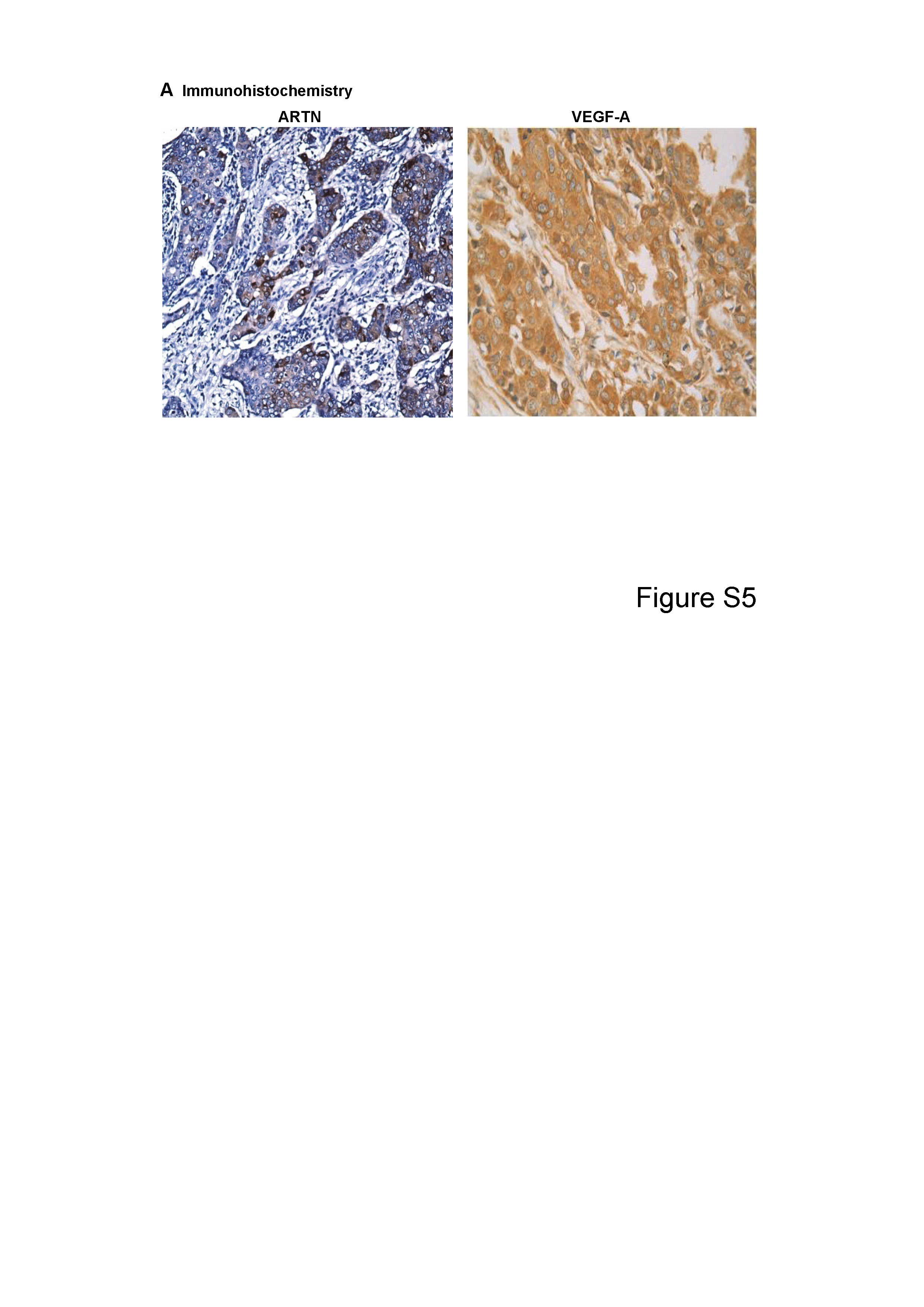

Supplement: Figure S5 — Immunohistochemistry (IHC) images of ARTN and VEGF-A expression in ER-MC. (A) ARTN and VEGF-A expression was detected by IHC analysis in ER-MC samples. Both ARTN and VEGF-A are predominantly localized to cytoplasm of carcinoma cells. X200 magnifications. (TIF) [file pone.0050098.s005.tif]
